# The efficacy of a standardized questionnaire in facilitating personalized communication about problems encountered in cancer genetic counseling: design of a randomized controlled trial

**DOI:** 10.1186/1471-2407-14-26

**Published:** 2014-01-15

**Authors:** Willem Eijzenga, Neil K Aaronson, Irma Kluijt, Grace N Sidharta, Daniela EE Hahn, Margreet GEM Ausems, Eveline MA Bleiker

**Affiliations:** 1 The Netherlands Cancer Institute, Dept. of Psychosocial Research and Epidemiology, Plesmanlaan 121, 1066 CX Amsterdam, The Netherlands; 2 The Netherlands Cancer Institute, Family Cancer Clinic, Plesmanlaan 121, 1066 CX Amsterdam, The Netherlands; 3 Division of Biomedical Genetics, University Medical Center Utrecht, Utrecht, The Netherlands

**Keywords:** Randomized controlled trial, Genetic counseling, Clinical genetics, Psychosocial problems, Hereditary cancers, Communication, Screening

## Abstract

**Background:**

Individuals with a personal or family history of cancer, can opt for genetic counseling and DNA-testing. Approximately 25% of these individuals experience clinically relevant levels of psychosocial distress, depression and/or anxiety after counseling. These problems are frequently left undetected by genetic counselors. The aim of this study is to evaluate the efficacy of a cancer genetics-specific screening questionnaire for psychosocial problems, the ‘Psychosocial Aspects of Hereditary Cancer (PAHC) questionnaire’ together with the Distress Thermometer, in: (1) facilitating personalized counselor-counselee communication; (2) increasing counselors’ awareness of their counselees’ psychosocial problems; and (3) facilitating the management of psychosocial problems during and after genetic counseling.

**Methods:**

This multicenter, randomized controlled trial will include 264 individuals undergoing cancer genetic counseling in two family cancer clinics in the Netherlands. Participants will be randomized to either: (1) an intervention group that completes the PAHC questionnaire, the results of which are made available to the genetic counselor prior to the counseling session; or (2) a control group that completes the PAHC questionnaire, but without feedback being given to the genetic counselor. The genetic counseling sessions will be audiotaped for content analysis. Additionally, study participants will be asked to complete questionnaires at baseline, three weeks after the initial counseling session, and four months after a telephone follow-up counseling session. The genetic counselors will be asked to complete questionnaires at the start of and at completion of the study, as well as a checklist directly after each counseling session. The questionnaires/checklists of the study include items on communication during genetic counseling, counselor awareness of their clients’ psychosocial problems, the (perceived) need for professional psychosocial support, cancer worries, general distress, specific psychosocial problems, satisfaction with care received, and experience using the PAHC questionnaire.

**Discussion:**

This study will provide empirical evidence regarding the efficacy of a relatively brief psychosocial screening questionnaire in terms of facilitating personalized communication, increasing counselors’ awareness, and optimizing management of psychosocial problems in the cancer genetic counseling setting.

**Trial registration:**

This study is registered at the Netherlands Trial Register (NTR3205) and ClinicalTrials.gov (NCT01562431).

## Background

Genetic counseling is offered to individuals who are at high risk of carrying a cancer gene mutation and who are at high risk of developing hereditary cancer. Factors related to *hereditary* cancer are: a cancer diagnosis at a young age, multiple relatives with a similar cancer diagnosis or a specific combination of cancers and a proven gene mutation in the family [1]. Reviews of previous studies indicate that, on average, genetic counseling does not have adverse psychological effects (i.e., depression, anxiety, distress). However, approximately 25% of high risk individuals experience clinically relevant adverse psychosocial effects after counseling [2-13].

It has been estimated that approximately one-third of counseless have some level of unmet need for psychosocial services in relation to genetic counseling [14,15]. This is not entirely surprising, in that genetic counselors focus primarily on gathering and communicating biomedical information, and often have a ‘teaching’ communication style [16]. This creates a situation where there is less time available to discuss potentially relevant psychosocial issues.

Patient reported outcome (PRO) measures have been used in a range of health care settings as a tool to improve communication between patients and their health care providers about relevant physical and psychosocial health problems [17-20]. Facilitating such communication has been hypothesized to have a cascade of effects, including improved provider awareness of their patients’ problems, improved patient care and management, including appropriate referrals, and ultimately, improved health outcomes [21-24].

Recently, we developed a psychosocial screening questionnaire specifically for the clinical cancer genetics setting, the Psychosocial Aspects of Hereditary Cancer (PAHC) questionnaire [25]. The PAHC questionnaire comprises: (1) 26 items organized into 6 problem domains (i.e., hereditary predisposition, family- and social issues, practical issues, general emotional issues, cancer-specific issues, and, for those who have children, children-specific issues), with response options ranging from 1 (‘not at all’) to 4 (‘very much’); (2) a question, per problem domain, about the desire to talk to a specialized psychosocial health professional; and (3) the Distress Thermometer (DT), a single item visual analogue scale ranging from 0–10, with 0 representing ‘no distress,’ and 10 ‘severe distress’ [26].

The aim of this randomized, controlled trial is to evaluate the efficacy of the PAHC questionnaire when used routinely in daily clinical cancer genetics practice in: (1) facilitating communication during genetic counseling sessions about relevant psychosocial issues; (2) increasing genetic counselors’ awareness of the psychosocial problems of their counselees; and (3) facilitating the appropriate management of these cancer genetic-specific psychosocial problems. Specifically, our primary research hypotheses are that the use of the PAHC questionnaire during genetic counseling will:

(1) increase significantly the number of psychosocial issues discussed during genetic counseling sessions;

(2) increase significantly the genetic counselors’ awareness of the psychosocial problems experienced by their counselees; and

(3) improve significantly the management of cancer genetic-specific psychosocial problems as evidenced by the referrals to psychosocial care and/or to sources of information about psychosocial issues. Additionally, we hypothesize that the routine use of the PAHC questionnaire will:

(4) increase significantly the number of discussed issues initiated by the counselor;

(5) increase significantly counselees’ satisfaction with the counseling process;

(6) decrease significantly counselees’ levels of cancer worry and distress during and after the genetic counseling process;

(7) decrease significantly the cancer genetic-specific problems experienced by the counselee after the genetic counseling process; and

(8) *not* increase significantly the total duration of the genetic counseling session.

## Methods/design

This is a prospective, multicenter, randomized controlled trial in which participants will be randomly allocated to: (1) an intervention group that completes the PAHC questionnaire prior to genetic counseling, the results of which are provided to the genetic counselor; or (2) a control group that completes the PAHC questionnaire, without feedback being given to the genetic counselor. The study consists of two phases. In the first part of the study, the focus is on the efficacy of the intervention during the first face-to-face genetic counseling session. The second phase of the study is concerned with the efficacy of the intervention during a telephone follow-up held approximately 4 weeks after DNA-test results are disclosed in a final face-to-face counseling session.

**Figure 1:**
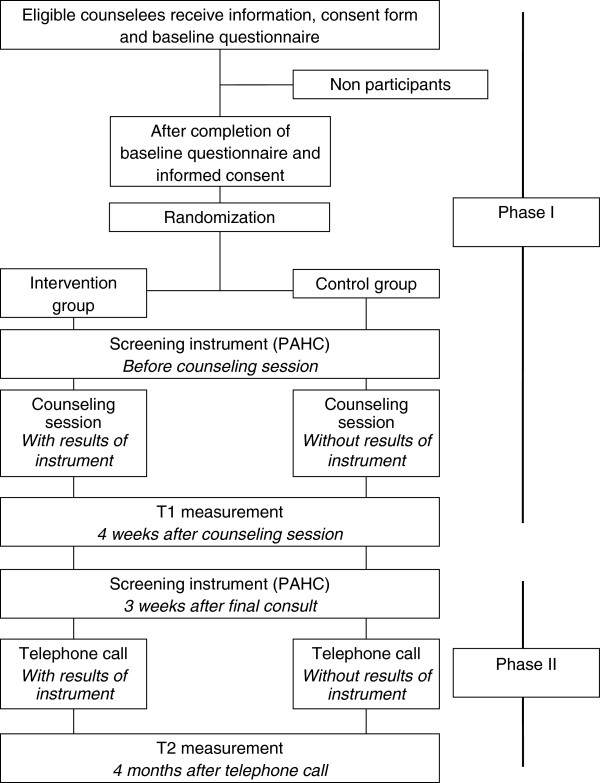
Design of the trial.

The primary outcome measures are counselor-counselee communication about psychosocial problems, counselors’ awareness of their counselees’ psychosocial problems, and improved management of those problems. Secondary outcomes include satisfaction with the counseling process, cancer worries, psychological distress, and prevalence of psychosocial problems. The design of the study and the anticipated flow of participants are displayed graphically in Figure 1.

The institutional review boards of The Netherlands Cancer Institute in Amsterdam, and the University Medical Center Utrecht have approved the study. This study follows the CONSORT guidelines [27] and is registered at the Netherlands Trial Register (NTR3205) and ClinicalTrials.gov (NCT01562431).

### Study sample

The study sample will be composed of 264 counselees who request genetic counseling at either The Netherlands Cancer Institute in Amsterdam or the University Medical Center Utrecht. Counselees will be excluded from the study if they are younger than 18 years of age, do not have basic fluency in the Dutch language or are participating in another study that would interfere with the current study.

### Recruitment and randomization

All eligible counselees will receive an invitation letter from the family cancer clinic, an informed consent form, a baseline questionnaire, and a return envelope three weeks before their first counseling session. Upon returning the completed informed consent form and the baseline questionnaire, we will randomize the participants on a 1:1 basis to either the intervention group or the control group. The minimization method will be used to balance the intervention and control group for each counselor in terms of gender and the cancer syndrome for which genetic counseling is requested [28]. Neither the counselees nor the counselors will be (or can be) blinded to group assigment.

### Intervention group procedure

Within the Netherlands, individuals seeking cancer genetic counseling routinely undergo a first consultation with a genetic counselor or a clinical geneticist and, when opting for a DNA-test, a final counseling session during which the DNA-test results are disclosed to the counselee. If indicated, screening recommendations for the patient and relatives are discussed within these sessions. The study intervention will take place within this standardized process, at the time of the first face-to-face genetic counseling session (phase I), and by telephone 4 weeks after the final face-to-face counseling session (phase II). The participants in the intervention group will be asked to complete the PAHC questionnaire either via the internet or, if preferred, by mail shortly before the face-to-face and the telephone follow-up counseling sessions. The counselee’s responses to the PAHC questionnaire will be made available to the genetic counselor prior to the counseling sessions.

The PAHC questionnaire consists of 26 questions addressing psychosocial problems and worries that are specifically relevant to counselees within the cancer genetics counseling and testing setting. The content of the PAHC questionnaire is organized into the following six domains: (1) hereditary predisposition; (2) family- and social issues; (3) practical issues; (4) general emotional issues; (5) cancer-specific issues; and, for those who have children 6) children-specific issues. The number of items per domain varies between 2 and 7. All 26 items are scored on a 4-point, Likert-type scale ranging from 1 (“not at all”) to 4 (“very much”). The PAHC questionnaire is supplemented by the Distress Thermometer (DT), a visual analogue scale ranging from 0–10 (no distress-severe distress). The timeframe of the PAHC questionnaire and the DT is the previous week. Per problem domain assessed by the PAHC questionnaire, the respondent is asked to indicate whether (s)he would like to receive professional psychosocial support [25].

The results of the PAHC questionnaire + DT will be printed and attached to the counselee’s medical record so that they are available to the genetic counselor prior to the relevant counseling session (face-to-face in phase I and telephone-based in phase II). To facilitate the genetic counselor’s rapid review of the questionnaire output, all problem domains for which the counselee responds “quite a bit” or “very much” to at least one item are color coded *red*, indicating a problem area that should preferably be discussed during the counseling session. All other problem domains, are color coded *green*, indicating that there is, in principle, no need to discuss it during the counseling. Additionally, based on the literature and analysis of a validation of the DT in a previous study by our group using a heterogeneous sample of counselees for cancer in the Netherlands, a score of 4 or greater on the DT is used to indicate a clinically relevant level of distress [25,29]. Finally, the counselor will receive information about whether the counselee is interested in obtaining additional professional psychosocial support for any given problem area. Scores on the DT above the cut-off point, and counselees’ requests for additional psychosocial support are also color coded *red.* Additionally, to increase the ease of interpretation of the results, all items above the threshold will be printed in a bold font, and those items that are not above the threshold will be printed in light-grey. All counselors will receive written instructions/guidelines and training in the use of the PAHC questionnaire and the DT.

### Control group procedure

Counselees in the control group will complete the PAHC questionnaire and DT as described above for the intervention group. However, the results of these questionnaires will not be provided to the genetic counselors.

### Timing and content of study measures

Counselees will be asked to complete questionnaires at: (1) baseline, prior to randomization; (2) approximately 4 weeks following the first genetic counseling session, before the final counseling session takes place; and (3) approximately 4 months after the telephone-based counseling session.

The counselors will be asked to complete a baseline questionnaire at the start of the study, a checklist at the end of each counseling session, and a final questionnaire at the end of the study. Both the in-person and the telephone-based genetic counseling sessions will be audiotaped by two independent raters (WE, GNS) for purposes of content analysis (see below). Inter-rater reliability will be assessed by double coding 10% of the audiotaped sessions, equally divided between the intervention group and control group sessions.

#### Sociodemographic and clinical data

The counselees’ age, gender, marital status, education level, number and age of children, and use of psychosocial services in the past and during the study, will be obtained via the self-report questionnaires (Table 1). Data on whether (s)he was diagnosed with cancer in the past and, if so, at what age, whether there is a known gene mutation in the family, the counselees’ genetic test results, and the number of genetic counseling sessions will be extracted from the medical records.

**Table 1:** Content and timing of study measures

	**Content of the measurement**	**Timing of measurement**
**Counselees**		
*Baseline (first) questionnaire*	Cancer worries	Before randomization
	General distress	
	Demographic data	
*Second questionnaire (T1)*	Evaluation of screening instrument	4 weeks after genetic counseling, before final consult, end of phase I
	Evaluation of counselor	
	General distress	
	Need for extra support	
	Cancer worries	
*Third questionnaire (T2)*	Specific psychosocial problems	4 months after the telephone call,
	Need for extra support	end of phase II
	Cancer worries	
	Evaluation of screening instrument	
	Satisfaction	
	General distress	
**Counselors**		
*Checklist*	Counselors’ awareness	After each counseling session and telephone call
*Baseline questionnaire*	Demographic data	Before the beginning of trial
*First questionnaire*	Evaluation of screening instrument	After finishing the trial
**Counselees and counselors**	Audio tapes	Genetic counseling
		Telephone call, 4 weeks after DNA-test disclosure

The counselors’ age, gender, and the number of years working at the family cancer clinic will be determined by questionnaire. (Table 1).

### Primary outcome measures

The primary outcomes of the trial are: (1) discussion of psychosocial problems; (2) counselors’ awareness of the counselees’ psychosocial problems; and (3) management of psychosocial problems during and after genetic counseling.

#### Discussion of psychosocial problems

Counselor-counselee communication about psychosocial issues will be assessed via content analysis of the audiotaped counseling sessions. Using a study-specific questionnaire, each counseling session will be coded for the specific psychosocial issues discussed during the counseling session. The coding reflects the 26 psychosocial issues of the PAHC questionnaire. Additionally, the percentage of counseling time devoted to the discussion of psychosocial issues will be calculated.

#### Counselors’ awareness

Counselors’ awareness of the psychosocial problems as experienced by their counselees will be assessed with a checklist completed by the counselors directly after the counseling sessions. The counselor will be asked to report whether (s)he believes that the counselee is experiencing problems in each of the 6 problem domains covered by the PAHC questionnaire on a 4-point scale ranging from (1, “no problem” to 4, “a severe problem”). The counselors’ ratings will be compared to the responses provided by the counselees on the PAHC questionnaire.

#### Management of psychosocial problems

The audiotapes of the counseling sessions will also be used to evaluate how the counselees’ psychosocial problems are managed. Specifically, the study-specific checklist will be used to code whether counselees were referred to additional sources of information about how to deal with psychosocial problems (e.g., websites or written materials) or to additional psychosocial counseling. The counselees will be asked to report their actual use of psychosocial services.

### Secondary outcome measures

Secondary outcomes include: (1) initiation of problem discussion; (2) the time devoted to discussing each psychosocial problem and the total duration of the counseling session; (3) cancer worries and general psychological distress; (4) cancer genetics-specific psychosocial problems; and (5) counselees’ and counselors’ satisfaction with the genetic counseling and with the intervention (the latter for the intervention group only).

#### Initiation of psychosocial issue discussion, and time devoted to such discussions

The audiotapes of the counseling sessions will be coded for who initiated the discussion of each specific psychosocial issue (i.e., the counselee or the counselor), the amount of time spent talking about psychosocial issues, and the total length of the counseling session.

#### Cancer worries and general psychological distress

Cancer worries will be assessed using an adapted version of the Cancer Worry Scale (CWS) as used in previous studies [14,15,30]. The CWS is an 8-item questionnaire measuring the frequency of cancer worries, the impact of worries on mood, and the impact of worries on daily functioning.

The Hospital Anxiety and Depression Scale (HADS) will be used to assess general psychological distress [31]. The HADS includes 14 questions and yields a total score, as well as subscale scores for anxiety and depression. It has been validated for use in the Netherlands [32].

#### Specific psychosocial problems

The PAHC questionnaire will be used to assess (changes over time in) specific psychosocial problems experienced by counselees in both the intervention and the control group. This will be evaluated at both the individual item as well as the problem domain level.

#### Satisfaction, evaluation and feasibility

Counselee and counselor satisfaction with both the genetic counseling itself and with the intervention (the latter for the intervention group only) will be assessed using an adapted version as used in a study by Bleiker et al. [33].

### Sample size and power calculations

We have based the sample size estimates on expected differences between the intervention group and the control group in communication between the genetic counselors and counselees about psychosocial issues. Overall power calculations for estimating sample size requirements were based on the following criteria for defining a substantively meaningful statistical association: (1) power of 0.80, (2) alpha of 0.05, and an (3) effect size “d” of 0.4. With these criteria, 99 cases per study arm are needed, resulting in a total sample size of 198 cases.

We anticipate that approximately 25% of the counselees will not have more than one counseling session and thus will not have a DNA-test disclosure session. Therefore, in order to have sufficient power in the second phase of the trial, we will include 264 participants at the start of the trial.

### Statistical analyses

All analyses will be performed on an intention-to-treat basis. Missing data on the HADS and CWS will be imputed using half-scale mean substitution methods. Data of participants who complete and return their first follow-up questionnaire after their final counseling session will be omitted from the analysis, because knowing the DNA-test result might influence questionnaire responses. Between rater agreement on the audiotaped sessions will be assessed by calculating the percentage of absolute agreement. Effect sizes will be calculated using standard statistical approaches.

#### Non-participant analysis

Based on experience with other studies, we anticipate that approximately 40% of eligible participants will decline to participate in the study. The non-participants will be compared with participants on available sociodemographic and clinical data using appropriate statistics (e.g. Student’s t-test, or non-parametric test).

#### Comparability of intervention and control group

The comparability of the intervention and control groups at baseline will first be evaluated in terms of sociodemographic and clinical characteristics. Student’s t-test or appropriate non-parametric tests will be used. If, despite the stratified randomization procedures, the groups are found to be statistically different on one or more baseline characteristics, these variables will be adjusted for in subsequent analyses.

#### Main research hypotheses

We will evaluate group differences in the number of psychosocial issues discussed during the genetic counseling sessions using analysis of (co)variance. We will assess counselors’ awareness of their counselees’ psychosocial problems by calculating the agreement between counselees’ and counselors’ on their ratings, per domain, of the psychosocial problems experienced by the counselees. We will calculate an Intraclass Correlation Coefficient (ICC2.1.A [34]) per domain for both the intervention and control group. Group differences will be assessed by treating the ICC’s as Pearson correlation coefficients and using Fisher’s r-to-z transformation to test for statistical differences per domain. Analysis of (co)variance will also be used to evaluate group differences in referral to additional information sources and/or referral to psychosocial care services, and the actual use of such services.

#### Secondary research hypotheses

We will employ analysis of (co)variance to evaluate group differences in the frequency of with which the genetic counselor initiated the discussion of psychosocial issues. Analysis of (co)variance will also be used to evaluate group differences in the amount of counseling time spent discussing psychosocial issues, and the total length of the counseling session. To evaluate group differences in cancer worries and general distress, we will use analysis of covariance, with the previous scores on these questionnaires as covariate. Logistic regression analysis will be used to evaluate differences between groups in the prevalence of cancer genetics-specific problems. Group differences in satisfaction with the genetic counseling process will be examined with Student’s t-tests and chi square tests, where appropriate. Intervention group and counselor satisfaction with the intervention will be reported descriptively.

## Discussion

Previous studies have shown that psychosocial problems experienced by individuals undergoing genetic counseling for cancer are often left undetected and thus untreated. One way that has been proposed to address this problem is to make use of patient reported outcome (PRO) measures in routine clinical practice to first identify, and then to manage relevant psychosocial issues. Previous studies that have evaluated the efficacy of implementing PRO measures in clinical settings have shown an increase in communication about health-related issues and an increase in clinicians’ awareness of their patients’ problems and, to a lesser extent an improvement in patient management and health over time [21-23,35]. To our knowledge, no previous studies have investigated the value of using such PRO data in daily clinical cancer genetic counseling.

This clinical trial will evaluate the efficacy of using a relatively brief, psychosocial screening questionnaire, the PAHC questionnaire, in improving communication about, recognition of, and management of psychosocial problems among individuals undergoing cancer genetic counseling and testing.

### Methodological issues

A major strength of the study is its use of a randomized design that will ensure high levels of internal validity. The relatively large sample size, the multicenter approach, and the heterogeneity of the study sample will increase the external validity and generalizability of the findings.

Several possible limitations of the study should also be noted. First, due to the nature of the intervention, it is not possible to blind the genetic counselors, nor the counselees, nor the raters of the audiotapes to group allocation. This carries with it the risk of contamination, particularly on the part of the counselors. That is, any given genetic counselor will be seeing both counselees assigned to the intervention group and the control group. Thus, it is conceivable that the counselors’ experience with the intervention (i.e., receiving personalized feedback regarding the counselees’ self-reported psychosocial problems) will also affect the way they interact with counselees in the conrol group. We would note, however, that any such carry over effect will have a conservative effect on the study (i.e., that, if anything, it will make it more difficult to observe significant group differences on the primary study outcomes). A second possible limitation of the study will depend on the observed prevalence of psychosocial problems among counselees. If the prevalence is low, then it will be more difficult to detect group differences in the various study outcomes. Finally, due to funding limits, the follow-up period for the second phase of the trial is relatively short (only 4 months).

## Conclusion

If proven efficacious, the introduction of a standardized procedure for assessing the psychosocial problems and needs of individuals undergoing cancer genetic counseling and testing will be a welcome addition to current clinical practice. It will facilitate timely discussion, detection and treatment of psychosocial issues specific to the cancer genetics setting. This is particularly important given the fact that the number of requests for genetic counseling is expected to continue to increase in the future. This will place additional demands on the time and resources of genetic counselors. Tools that facilitate early detection and treatment, or referral of those with specific psychosocial problems and concerns, are welcome.

## Competing interests

The authors declare that they have no competing interests.

## Authors’ contributions

NKA, IK, DEEH, and EMAB are the principal investigators of this study. WE is the PhD student on this study, and generated the first draft of this manuscript based on the study protocol. MGEMA is the clinical geneticist at University Medical Center Utrecht. GNS is the research assistant on this study. All authors approved the final version of the manuscript.

## Pre-publication history

The pre-publication history for this paper can be accessed here:

http://www.biomedcentral.com/1471-2407/14/26/prepub
